# Neurodevelopmental Phenotype Associated with *TRIP12*: Report of a Family Carrying the p.Asp1135Val Variant

**DOI:** 10.3390/genes16121456

**Published:** 2025-12-05

**Authors:** Katia Margiotti, Marco Fabiani, Antonella Cima, Francesca Monaco, Antonella Viola, Alvaro Mesoraca, Claudio Giorlandino

**Affiliations:** 1 Human Genetics Lab, Altamedica Main Centre, Viale Liegi 45, 00198 Rome, Italy; marco.fabiani@artemisia.it (M.F.);; 2 Department of Prenatal Diagnosis, Altamedica Fetal-Maternal Medical Centre, Viale Liegi 45, 00198 Rome, Italy

**Keywords:** *TRIP12* gene, autism spectrum disorder (ASD), whole-exome sequencing (WES), intellectual disability, speech disorder

## Abstract

Background: Pathogenic variants in the *TRIP12* gene are associated with Clark-Baraitser syndrome, a condition characterized by neurodevelopmental disorders, including intellectual disability, autism spectrum disorder (ASD), and speech delay. Phenotypic expression is variable, and facial features are not consistently present. Familial inheritance is rare. Methods: Whole-exome sequencing (WES) was performed on a proband with speech disorder and ASD, as well as on her parents. Clinical assessment included developmental, cognitive, and physical evaluations. Results: A heterozygous missense variant c.3404A>T (p. Asp1135Val) in the *TRIP12* gene was identified in both the proband and her father. Both presented with speech disorder and ASD without facial features or severe intellectual disability. Conclusions: In line with recent genotype–phenotype studies, missense TRIP12 variants tend to be associated with milder neurodevelopmental presentations, typically characterized by mild to moderate intellectual impairment, variable autistic traits, limited or absent facial features, and a low incidence of epilepsy. This familial case further presents the phenotypic spectrum of *TRIP12* missense variants and highlights that ASD and speech disorder may occur as isolated neurodevelopmental findings without syndromic features. The report reinforces the relevance of *TRIP12* analysis in the differential diagnosis of ASD and language disorders, even in individuals lacking physical traits, supporting more accurate genetic counseling and broader awareness of inherited TRIP12-related conditions.

## 1. Introduction

The *TRIP12* gene (Thyroid hormone receptor-interacting protein 12, OMIM 604506), located on chromosome 2q36.3, encodes an E3 ubiquitin ligase belonging to the HECT (Homologous to E6-AP C-Terminus) family [1]. This enzyme plays a key role in the ubiquitin–proteasome pathway, which is fundamental for maintaining protein homeostasis and regulating essential processes such as cell cycle progression, DNA damage response, chromatin remodeling, and cell differentiation [2,3]. Pathogenic variants in the *TRIP12* gene disrupt E3 ligase activity, with downstream effects on neurodevelopmental processes. These variants were first linked to Clark–Baraitser syndrome (OMIM #617752), initially described in 1987 in a mother and two sons with intellectual disability (ID), obesity, and facial features [3,4].

Subsequent studies have shown that de novo or inherited *TRIP12* variants cause variable intellectual disability, autism spectrum disorder (ASD), epilepsy, and sometimes dysmorphic traits [5,6]. Intellectual disability is reported in 100% of individuals with pathogenic *TRIP12* variants, with cognitive performance ranging from mild to severe [2]. Among individuals carrying pathogenic or likely pathogenic variants in the *TRIP12* gene, around half present with autistic features, supporting the classification of *TRIP12* as a high-confidence autism spectrum disorder (ASD) gene in resources such as SFARI (Simons Foundation Autism Research Initiative), which ranks genes based on the strength of evidence linking them to ASD [7,8]. A hallmark feature of the *TRIP12*-associated neurodevelopmental phenotype is speech and language impairment, often coexisting with apraxia of speech (CAS) [9]. In one cohort of 17 probands with pathogenic *TRIP12* variants, 88% had expressive language difficulties, ranging from single words to a complete absence of verbal language [9]. The clinical phenotype associated with *TRIP12* shows marked variability. While some patients present with recognizable facial features reminiscent of Clark–Baraitser syndrome, others show no obvious physical features [5]. Inherited variants are associated with milder phenotypes; pathogenic variants inherited from an affected parent (often with mild intellectual disability) may present with more severe manifestations in offspring [5]. At the molecular level, *TRIP12* contains a catalytic HECT domain essential for ubiquitin ligase activity, as well as WWE and ARM domains that mediate protein–protein interactions. Missense variants affecting critical regions (e.g., within the HECT domain) may reduce protein stability or alter enzymatic activity, often correlating with more severe phenotypes, including regression and lower cognitive and language outcomes [1]. This report describes a familial case of the c.3404A>T; p.Asp1135Val (NM_004238.3; rs778262518) *TRIP12* variant in a father and daughter, both presenting with speech impairment and a diagnosis of ASD (DSM-5 severity level 1), but without dysmorphic features. This case further broadens the understanding of the *TRIP12*-associated clinical spectrum, highlighting the phenotypic variability, absence of classic facial features in some familial cases, and the significance of inherited variants.

## 2. Case Presentation

**Figure 1:**
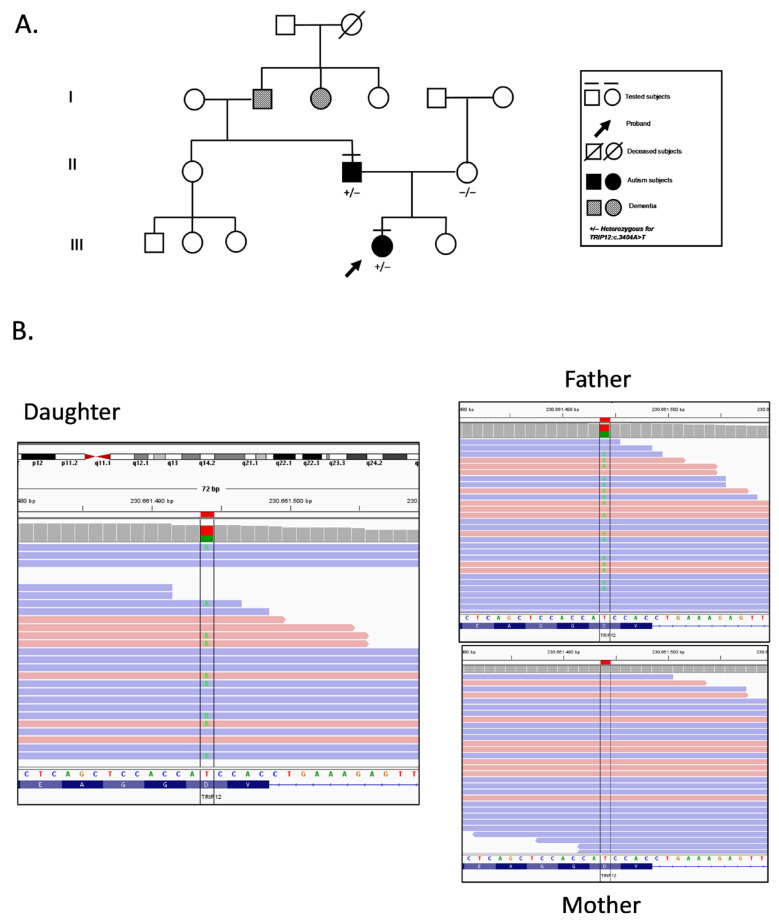
**Familial segregation of the *TRIP12* variant.** (**A**) Pedigree of the family carrying the heterozygous *TRIP12*:c.3404A>T (p.Asp1135Val) variant. The proband (arrow) and her father are both heterozygous carriers and clinically diagnosed with Autism Spectrum Disorder (ASD, DSM-5 severity level 1). The mother tested negative for the variant. Deceased subjects are indicated with a diagonal line. Individuals affected by dementia are indicated in the pedigree. Tested individuals are marked with “+/−” (heterozygous) or “−/−” (wild-type). (**B**) Integrative Genomics Viewer (IGV) visualization of aligned sequencing reads for each family member. The green color marks the position of the c.3404A>T substitution. The proband and her father show a heterozygous A/T signal at the variant site, whereas the mother carries only the reference A allele.

The proband (21-year-old female) presented with a history of speech disorder and features consistent with Autism Spectrum Disorder (ASD), without facial features or severe intellectual disability. Psychological assessment revealed difficulties in reciprocal social interaction, restricted interests, and avoidance behaviors. Personality evaluation confirmed introversion, social discomfort, and emotional withdrawal. Her father (51-year-old male) exhibited a similar neurobehavioral profile, with lifelong speech difficulties, social avoidance, and milder ASD traits (Figure 1). The diagnosis of ASD in both the proband (ADOS-2 score = 11) and her father (ADOS-2 score = 9) was established according to DSM-5 criteria, integrating clinical observation with standardized neuropsychological assessment tools [10]. Neither presented with congenital malformations, abnormal growth patterns, or distinct facial dysmorphism. However, anthropometric data and photographs could not be included as the family did not authorize publication of identifiable clinical information; nonetheless, specialist evaluation confirmed the absence of notable physical or facial anomalies. The family history revealed two paternal relatives who developed dementia at an advanced age, although no genetic testing was performed on these individuals (Figure 1). Both the proband and her father underwent genetic testing to investigate possible underlying causes of their condition, and the proband’s mother was also tested to assess variant segregation within the family. Trio-based whole-exome sequencing (WES) was performed, along with chromosomal microarray analysis (array-CGH), on DNA extracted from peripheral blood of all three individuals. WES, covering approximately 19,000 genes, identified a heterozygous *TRIP12* c.3404A>T (p.Asp1135Val; rs778262518) variant in both the proband and her father, while the mother tested negative, supporting paternal inheritance (Figure 1).

Only variants of uncertain significance (VUS) with a pathogenicity score ≥ 3.5 were considered for further evaluation. This threshold, based on eVai’s integrated ACMG/AMP criteria scoring system, is associated with an expected sensitivity > 96% and specificity > 99%, and reflects a higher likelihood of reclassification toward pathogenicity pending additional clinical or functional evidence [11]. Array-CGH excluded pathogenic copy number variations (CNVs). No other variants meeting ACMG/AMP criteria for pathogenicity, likely pathogenicity, or high-scoring variants of uncertain significance (VUS) related to the investigated Human Phenotype Ontology (HPO) terms including HP:0001249 (Intellectual disability), HP:0000717 (Autism), HP:0001263 (Global developmental delay), HP:0000750 (Delayed speech and language development), HP:0011098 (Speech apraxia), and HP:0002546 (Incomprehensible speech) were detected [12].

FMR1 testing for Fragile X syndrome was also negative. In the context of p.Asp1135Val variant interpretation, *TRIP12* demonstrates strong evidence of intolerance to both missense and loss-of-function variation. As of August 2025, no records in ClinVar and no prior peer-reviewed case reports describe this exact variant associated with phenotype; it is present only in population datasets (ExAC/EVA 2015; gnomAD/TopMed/ALFA updates through 2024) at very low allele frequency (gnomAD v2.1.1: 7/244,660; AF = 2.86 × 10^−5^; 0 homozygotes). According to gnomAD constraint metrics, the missense z-score of 6.366 indicates marked depletion of this missense variant. The pLI score of 1 (LOEUF = 0.145) confirms high intolerance to loss-of-function. These constraint metrics, combined with segregation of the variant in two affected family members, align with a likely pathogenic classification according to ACMG and AMP criteria, pending functional confirmation [13]. Structural assessment shows that p.Asp1135Val is located in exon 2 within a well-structured region of the TRIP12 protein, with a per-residue pLDDT confidence score above 90 in the AlphaFold model, indicating high reliability of the predicted local conformation. This residue is situated outside both the WWE domain (residues 759–847) and the HECT catalytic domain (residues 1712–1992). Pathogenicity prediction tools further support the relevance of this variant: AlphaMissense assigns a score of 0.967 to p.Asp1135Val, consistent with a high predicted pathogenic potential. The variant results in the replacement of a negatively charged aspartic acid residue with a hydrophobic valine residue within a structured region of the protein. This substitution can disrupt local electrostatic interactions or hydrogen bonding networks and may alter protein stability or conformational dynamics. AlphaMissense is a computational model that integrates sequence conservation, structural environment of the residue, and physicochemical changes introduced by the mutation to estimate the probability of pathogenicity. Evolutionary conservation of the Asp1135 residue was evaluated through phylogeny-based metrics integrated in the annotation pipeline, including PhyloP, PhastCons and GERP++, all of which indicate that this position is highly conserved across vertebrate species [11]. However, clinical correlation and functional assays will be required to clarify its impact on E3 ligase activity and neurodevelopmental phenotypes. In summary, whole-exome sequencing detected approximately 36,000 variants per individual; among these, the heterozygous p.Asp1135Val variant in the *TRIP12* gene was the only one that met the clinical relevance criteria in both the father and the daughter.

## 3. Discussion

Missense *TRIP12* variants, particularly those located in the HECT catalytic domain, have been recurrently associated with disruption of E3 ligase activity, impairing ubiquitin-mediated protein degradation and leading to neurodevelopmental consequences.

Based on variants reported in the HGMD, ClinVar and DECIPHER databases as of November 2025, the majority of pathogenic *TRIP12* alterations are loss-of-function (LoF), while missense variants represent a smaller proportion of the overall mutational spectrum. Pathogenic missense changes tend to cluster within functionally constrained regions, most notably the WWE and HECT domains, highlighting their relevance to TRIP12 protein function. Across all variant classes, the most recurrent clinical features include intellectual disability, autism spectrum disorder, developmental delay and marked speech impairment, consistent with the core neurodevelopmental phenotype associated with TRIP12 dysfunction.

Functional hypotheses suggest that such alterations can reduce protein stability, disrupt substrate interactions, or alter enzymatic turnover, ultimately affecting neuronal maturation, synaptic homeostasis, and transcriptional regulation. Nobakht et al. (2025) further propose that reduced protein stability may trigger a compensatory upregulation of *TRIP12* mRNA levels, partially mitigating the biochemical impact and explaining milder phenotypic presentations in some cases [14].

Familial *TRIP12* variants often manifest as milder, non-syndromic phenotypes compared to de novo variants. Aerden et al. (2023), in the largest cohort described to date, confirmed that inherited missense variants tend to present with less severe intellectual impairment, absence of epilepsy, and frequent lack of craniofacial dysmorphism, although subtle facial features may occasionally be observed [5]. This variability is reflected in our family, where both affected individuals presented with ASD and speech disorder without facial features. The current case parallels previous reports by Bramswig et al. (2017) and Donoghue et al. (2020), in which language delay and ASD were cardinal findings across different *TRIP12* genotypes, with physical features absent or mild in some inherited cases [2,6]. Our identification of the p.Asp1135Val variant in two affected relatives, both showing similar but relatively mild neurodevelopmental manifestations, supports the view that certain *TRIP12* variants can result in an attenuated, familial form of the disorder [2,6].

Zhang et al. (2017) originally described *TRIP12* haploinsufficiency as a cause of intellectual disability and ASD, highlighting variable expressivity even within the same family [1]. This variability likely reflects a combination of consequences (missense versus truncating variants, domain affected), compensatory mechanisms (e.g., mRNA upregulation), and possible modifying genetic or environmental factors.

Given the extensive genetic heterogeneity that characterizes neurodevelopmental disorders, including global developmental delay, intellectual disability and autism spectrum disorder, and the considerable clinical overlap among these conditions, the diagnosis of TRIP12-related syndrome cannot rely on single-gene tests. Even when suggestive clinical features are present, exome sequencing or an exome-based neurodevelopmental panel represents the most appropriate diagnostic approach, as it allows the identification of *TRIP12* variants within a highly complex genetic landscape.

Our case also emphasizes the importance of constraint metrics in variant interpretation: the high missense z-score (6.366), pLI = 1 (LOEUF 0.145), and extremely low pRec (5.6379 × 10^−38^) and pNull (1.397 × 10^−64^) provide strong evidence of intolerance to missense and LoF variation, further supporting pathogenicity under ACMG/AMP criteria. To our knowledge, this report represents the first description of *TRIP12* c.3404A>T (p.Asp1135Val; rs778262518) associated with a neurodevelopmental phenotype.

Overall, this case expands the phenotypic spectrum of *TRIP12*-associated disorders by reinforcing that missense variants can present in a familial form with ASD and speech disorder, without physical features. Such findings are crucial for genetic counseling, as they underline the need to consider *TRIP12* analysis even in patients without the classic Clark–Baraitser facial features, particularly when there is familial recurrence of ASD or language impairment.

## 4. Conclusions

This is, to the best of our knowledge, the first report linking *TRIP12* c.3404A>T (p.Asp1135Val; rs778262518) to a neurodevelopmental phenotype (ASD and speech disorder). These findings warrant deposition and public sharing of this variant with phenotypic details to facilitate future case aggregation and curation. This familial case of the TRIP12 p.Asp1135Val (rs778262518) missense variant expands the clinical spectrum of TRIP12-associated neurodevelopmental disorders. Both affected individuals presented with speech disorder and ASD without facial features, consistent with the milder phenotype often observed in inherited missense variants [2]. The absence of facial features underscores the phenotypic variability of *TRIP12* variants and supports the inclusion of *TRIP12* in the genetic evaluation of ASD and language disorders. Recognition of such cases is important for genetic counseling, as familial *TRIP12* variants may confer a risk for recurrence of mild neurodevelopmental phenotypes without syndromic presentation. Continued aggregation of familial cases and functional characterization of variants will be essential to clarify genotype–phenotype correlations and refine prognostic counseling.

## Data Availability

The original data presented in the study are openly available in ClinVar at SCV006304771.1.
